# miR-30a-3p participates in the development of asthma by targeting CCR3

**DOI:** 10.1515/med-2020-0102

**Published:** 2020-06-02

**Authors:** Xiaobo Li, Binliang Wang, Mao Huang, Xiaomi Wang

**Affiliations:** Department of Respiratory and Critical Care Medicine, Taizhou First People’s Hospital, Taizhou 318020, P. R. China

**Keywords:** miR-30a-3p, CCR3, ovalbumin, asthma, eosinophils

## Abstract

This study aimed to investigate the role and relevant mechanism of miR-30a-3p action in asthma. The results of this study revealed that the expression levels of miR-30a-3p were significantly decreased in the peripheral blood of asthmatic patients. In addition, we found that the CC chemokine receptor (CCR3) was a target of miR-30a-3p. Subsequently, an asthma mouse model was established using ovalbumin (OVA). The results showed that the expression of miR-30a-3p and CCR3 was downregulated and upregulated, respectively, in the peripheral blood of asthmatic mice. Enzyme-linked immunosorbent assay (ELISA) in asthmatic mouse serum demonstrated that miR-30a-3p mimic treatment significantly decreased the secretion of OVA-specific IgE, eotaxin-1, interleukin (IL)-5, and IL-4. These results suggested that miR-30a-3p inhibited CCR3 signaling pathway and relieved the inflammatory response against asthma *in vivo*. Eosinophils have also been implicated in the asthmatic inflammatory response. Therefore, the *in vitro* effects of miR-30a-3p on eosinophil activity were determined. Findings suggested that miR-30a-3p mimic significantly reduced eosinophil viability and migration and induced apoptosis. In addition, CCR3 and eotaxin-1 downregulation were observed. The aforementioned results were significantly reversed following CCR3 overexpression. This study suggested that miR-30a-3p was involved in asthma by regulating eosinophil activity and targeting CCR3.

## 1 Introduction

Asthma is a very common chronic inflammatory disease and a leading cause of morbidity in children and adults worldwide [1,2]. The main clinical features of asthma are wheezing, shortness of breath, chest tightness, and cough. The frequency and severity of these features may change over time [3]. The incidence of asthma in different countries ranges and affects 1–18% of the general population, whereas it is estimated that approximately 300 million people worldwide suffer from this disease [4]. The increasing incidence and mortality of asthma that has been noted in recent years are possibly attributed to its complex pathogenesis. Bousquet et al. demonstrated that eosinophilia was associated with the severity of asthma [5]. Therefore, blood eosinophil counts provide a readily available multifunctional biomarker for severe eosinophilic asthma [6,7].

MicroRNAs (miRNAs) are small (∼22 nucleotides) and highly conserved regulatory noncoding RNAs that inhibit gene expression by translational repression or mRNA transduction [8]. MiRNAs regulate gene expression on the posttranscriptional level via targeting the 3′-untranslated region (3′-UTR) of target mRNAs to promote mRNA degradation or inhibit protein translation [9]. Accumulating evidence has demonstrated that miRNAs participate in the development and progression of human cancers [10,11,12]. It has been reported that miRNAs regulate asthma pathogenesis and are potential targets for the treatment of the disease. Malmhäll et al. showed that miR-155 knockdown resulted in diminished eosinophilic inflammation and mucus secretion in the lungs of asthmatic mice [13]. In addition, Collison et al. demonstrated that miR-145 downregulation inhibited eosinophilic inflammation, mucus hypersecretion, and the production of type 2 (Th2) cytokines [14]. MiR-30a-3p is a member of the evolutionarily conserved miR-30a family [15] and it has been reported to participate in the Wnt signaling pathway in breast cancer, multiple myeloma, and glioma [16,17,18]. Although the role of miR-30a-3p in several types of cancer, including hepatic, lung, and cervical cancer [19,20,21], has been extensively investigated, its role in asthma remains unclear.

The CC chemokine receptor 3 (CCR3) is functionally expressed on eosinophils [22,23]. Several studies revealed that CCR3 downregulation inhibited eosinophil recruitment in an acute model of asthma [24,25,26]. Shen et al. showed that CCR3 monoclonal antibody significantly inhibited airway eosinophilia and mucus overproduction in asthmatic mice, indicating that blockage of CCR3 may represent a new strategy for asthma treatment [27]. Interestingly, we found through bioinformatics analysis that CCR3 is a potential target gene for miR-30a-3p. Therefore, we hypothesized that miR-30a-3p may play an important role in asthma progression by regulating the expression of CCR3.

Therefore, the aim of this study was to investigate the expression and mechanism of miR-30a-3p in the development of asthma.

## 2 Materials and methods

### 2.1 Clinical specimen collection

Peripheral blood samples were collected from 30 asthmatic patients (age range: 19–57 years old; 15 man, 15 female) and 30 healthy volunteers (age range: 22–61 years old; 15 man, 15 female) at the Taizhou First People’s Hospital between May 2017 and December 2018. Inclusion criteria for asthma patients: asthma was diagnosed in line with the Global Initiative for Asthma (GINA) [28] with bronchodilation FEV1 change >200 mL and 12% or methacholine PC20 <2.5 mg; allergic asthma; nonacute attack; patients had not received any corticosteroid treatment in the last 3 months; patients were successfully induced sputum, of which 22 cases were eosinophilic (sputum eosinophils ≥3% and sputum neutrophils <61%) and 8 cases were neutrophilic (sputum neutrophils ≥61% and sputum eosinophils <3%). Exclusion criteria: acute episode; pregnancy; respiratory infection in the last 2 weeks; bronchiectasis; other respiratory disease; and serious organ failure. Healthy controls had no history of chronic respiratory disease. Written informed consent was obtained from every patient prior to the initiation of the study. This study was approved by the institutional ethics committee of the Taizhou First People’s Hospital.

### 2.2 qRT-PCR assay

Total RNA was extracted using Trizol reagent (Takara) according to the manufacturer’s instructions. All procedures were carried out on ice. Following RNA extraction, the concentration of each sample was measured using an ultraviolet spectrophotometer. Subsequently, cDNA was synthesized using a reverse transcription kit (Vazyme) according to the manufacturer’s instructions. The reaction conditions were as follows: 70°C for 5 min, 37°C for 5 min, and 42°C for 60 min. Finally, qPCR was performed using the SYBR kit (Vazyme) under the following conditions: 95°C for 3 min, 40 cycles of 95°C for 30 s, 56°C for 30 s, and 72°C for 30 s. The estimation of the *GAPDH* or *U6* expression levels served as an internal control for normalization. Primer sequences were listed as following:

miR-30a-3p forward: 5ʹ-CCCTGCTCTGGCTGGTCAAACGGA-3ʹ;
Reverse: 5ʹ-TTGCCAGCCCTGCTGTAGCTGGTTGAAG-3ʹ;
U6 forward: 5′-GCTTCGGCAGCACATATACTAAAAT-3′;
Reverse: 5′-CGCTTCACGAATTTGCGTGTCAT-3′;
GAPDH forward: 5′-CTTTGGTATCGTGGAAGGACTC-3′;
Reverse: 5′-GTAGAGGCAGGGATGATGTTCT-3′;
CCR3 forward: 5′-CCAGCTGTCAGCAGAGTAAA-3′;
Reverse: 5′-CTCACCAACAAAGGCGTAGA-3′;
Eotaxin-1 forward: 5′-TGAAGCTTGGGCCTTCTGTCCCAACC-3′;
Reverse: 5′-GGTCGACTGGAGTGAGATTTTTGGTC-3′. Gene expression was calculate by using the 2^−ΔΔCq^ method [29].

### 2.3 Western blot analysis

Total proteins were extracted from eosinophils using RIPA lysis buffer (Beyotime Institute of Biotechnology) supplemented with protease inhibitors. The extracted proteins were quantified with a bicinchoninic acid (BCA) assay kit (Beyotime Institute of Biotechnology). A total of 20 µg of protein was heated at 100°C for 5 min prior to loading, separated by 10% SDS–PAGE and subsequently transferred to a PVDF membrane (Merck KGaA). Following blocking for 1.5 h at room temperature with TBS containing 0.1% Tween and 5% fat-free powdered milk, the membranes were incubated overnight at 4°C with the primary antibodies anti-CCR3 (Cat no. Ab32512; 1:1,000; Abcam), anti-eotaxin-1 (Cat no. Ab25086; 1:1,000; Abcam), and GAPDH (Cat no. Ab181602; 1:1,000; Abcam). Following incubation with the primary antibody, the membranes were subsequently incubated at 4°C overnight with a secondary antibody (Cat no. 7074; 1:2,000; CST). The protein bands were detected using the enhanced chemiluminescence method (ECL; EMD Millipore). GAPDH served as the loading control for normalization of the protein levels.

### 2.4 Dual-luciferase reporter assay

The target genes of miR-30a-3p were predicted using the TargetScan Release 7.2 (www.targetscan.org/vert_72) bioinformatic tool. The bioinformatic analysis revealed that miR-30a-3p directly targeted the 3′-UTR of CCR3. The wild-type (CCR3-WT) and mutant (CCR3-MUT) 3′-UTR of CCR3 containing the putative binding site of miR-30a-3p were synthesized and respectively cloned into a pmiR-RB-Report™ dual luciferase reporter gene plasmid vector (Guangzhou RiboBio Co., Ltd). Subsequently, eosinophils were co-transfected with CCR3-WT or CCR3-MUT and miR-30a-3p mimic or mimic control using Lipofectamine^®^ 3000 (Invitrogen; Thermo Fisher Scientific, Inc.) for 48 h. Finally, the relative luciferase activity was detected using a Dual Luciferase Reporter Assay System (Promega Corporation) according to the manufacturer’s instructions.

### 2.5 Establishment of asthmatic murine model

A total of 40 male BALB/c mice (weight, 20–30 g; age, 6–8 weeks) were obtained from the Wenzhou Medical University Experimental Animal Center (Wenzhou, China). All mice were housed in a 12 h dark/light cycle at 25 ± 5°C and were provided *ad libitum* access to food and water. Animal experiments were performed according to the Recommended Guideline for the Care and Use of Laboratory Animals issued by the Chinese Council on Animal Research. This study was approved by the Animal Ethics Committee of the Wenzhou Medical University.

The mouse asthmatic model was established using OVA as previously described [30,31]. Briefly, mice were adaptively fed for 7 days, and then the hind legs were subcutaneously injected with 0.2 mL of normal saline containing 0.1 mg of OVA (Sangon Biotech Co., Ltd, Shanghai, China) and 10 mg of aluminum hydroxide (Beijing Solarbio Science & Technology Co., Ltd, China). The second sensitization injection was applied on day 14. For three weeks, starting on day 25, an airway challenge was performed daily for 30 min using aerosolized 1% OVA normal saline. Mice in the control group were only injected with the same amount of normal saline. The mice were divided into four groups (*n* = 10), namely the control, ovalbumin (OVA), OVA + mimic control and OVA + miR-30a-3p mimic group. In the OVA + mimic control and OVA + miR-30a-3p mimic groups, mice were administered with 40 µL of 20 µg mimic control or miR-30a-3p mimic by nasal instillation every three days for a total of 10 times starting on the day 20 [31].

During the experiment, the health and behavior (diet, drinking, tail swing) of all mice were monitored every 2 days. No mouse died during the experiments. When the mice lost >15% of their body weight (body weight prior to injection), the experiment was ended. After treatment, mice were anesthetized with pentobarbital (40 mg/kg) by intraperitoneal injection and sacrificed through cervical dislocation (death defined as the lack of heartbeat and breathing). The blood samples were subsequently harvested following euthanasia.

### 2.6 Eosinophil isolation and culture

Eosinophils were isolated from murine bone marrow of the control group using Percoll density gradient separation and the CD16 magnetic bead negative selection system (Miltenyi Biotec GmbH). Eosinophils were cultured in RPMI 1640 medium (Invitrogen; Thermo Fisher Scientific, Inc.) supplemented with 10% FBS (Invitrogen; Thermo Fisher Scientific, Inc.) and 1% (v/v) penicillin–streptomycin solution (Gibco; Thermo Fisher Scientific, Inc.) in a humidified atmosphere containing 5% CO_2_ at 37°C.

### 2.7 Cell transfection

Eosinophils were transfected for 48 h with 50 nM miR-30a-3p mimic (5′-CUUUCAGUCGGAUGUUUGCAGC-3′; GeneChem, Shanghai, China), 50 nM mimic control (5′-AAGGCAAGCUGACCCUGAAGU-3′; GeneChem, Shanghai, China), 1 µg CCR3-plasmid (Cat no. sc-419704-ACT; Santa Cruz Biotechnology, Inc.), 1 µg control-plasmid (Cat no. sc-418922; Santa Cruz Biotechnology, Inc.), 50 nM miR-30a-3p mimic + 1 µg control-plasmid or 50 nM miR-30a-3p mimic + 1 µg CCR3-plasmid using Lipofectamine^®^ 3000 (Invitrogen; Thermo Fisher Scientific, Inc.) according to the manufacturer’s instructions.

### 2.8 CCK-8 assay

Cell viability was determined by the CCK-8 assay (Dojindo Molecular Technologies, Inc.). Eosinophils were seeded onto a 96-well plate at a density of 3 × 10^3^ cells/well and were subsequently transfected with miR-30a-3p mimic, mimic control, miR-30a-3p mimic + control-plasmid, or miR-30a-3p mimic + CCR3-plasmid for 48 h. Finally, 10 µL CCK-8 reagent was added into each well and the cells were incubated at 37°C for an additional 4 h time period. The optical density (OD) values at 450 nm were read using a microplate reader.

### 2.9 Cell migration assay

A 24-well transwell plate (pore size, 8 µm) was used for cell migration assay. A total of 1 × 10^5^ cells were resuspended in 200 µL serum-free medium and placed in the upper chamber. RPMI 1640 medium (500 µL) containing 10% FBS was added to the bottom chamber. The cells in the 24-well plate were incubated for 24 h at 37°C in the presence of 5% CO_2_. Following incubation, the cells that had not migrated from the upper to the lower chamber were gently wiped away with a clean cotton swab. The cells on the lower chamber were fixed with 4% polyoxymethylene and stained with 0.1% crystal violet (both from Beyotime Institute of Biotechnology) for 20 min at room temperature. The cells that had migrated were counted with an optical microscope in five randomly selected fields.

### 2.10 Flow cytometry analysis

The apoptotic rate was analyzed by flow cytometry following eosinophil transfection with miR-30a-3p mimic, mimic control, miR-30a-3p mimic + control-plasmid, or miR-30a-3p mimic + CCR3-plasmid for 48 h. Subsequently, the cells were grown in 6-well plates, digested with trypsin, resuspended in fresh medium and centrifuged at 1,000 rpm for 5 min at room temperature. Finally, the cells were incubated with 5 µL Annexin V-FITC and 5 µL PI (Annexin V-FITC Apoptosis Detection Kit; Beyotime Institute of Biotechnology) at 4°C for 10 min in the dark. The induction of cell apoptosis was analyzed using flow cytometry (BD Biosciences) and the data were used to determine the percentage of apoptotic cells.

### 2.11 Enzyme-linked immunosorbent assay (ELISA)

The expression levels of OVA-specific IgE (Cat no. 439807; Biolegend), IL-5 (Cat no. PI602; Beyotime Institute of Biotechnolog), IL-4 (Cat no. PI612; Beyotime Institute of Biotechnolog), and eotaxin-1 (Cat no. Ab100680; Abcam) in the sera of asthmatic and healthy mice were assessed using specific ELISA kits according to the manufacturer’s instructions.

### 2.12 Statistical analysis

The data are presented as the mean ± standard deviation (SD) from at least three independent experiments. All statistical analyses were performed using the GraphPad Prism 6.0 software. The Student’s *t*-test was used to analyze the differences between two groups, whereas statistical differences among multiple groups were analyzed by one-way analysis of variance (ANOVA) followed by Tukey’s test. *p* < 0.05 was considered to indicate a significant difference.

## 3 Results

### 3.1 MiR-30a-3p is downregulated in asthmatic patients

**Figure 1:**
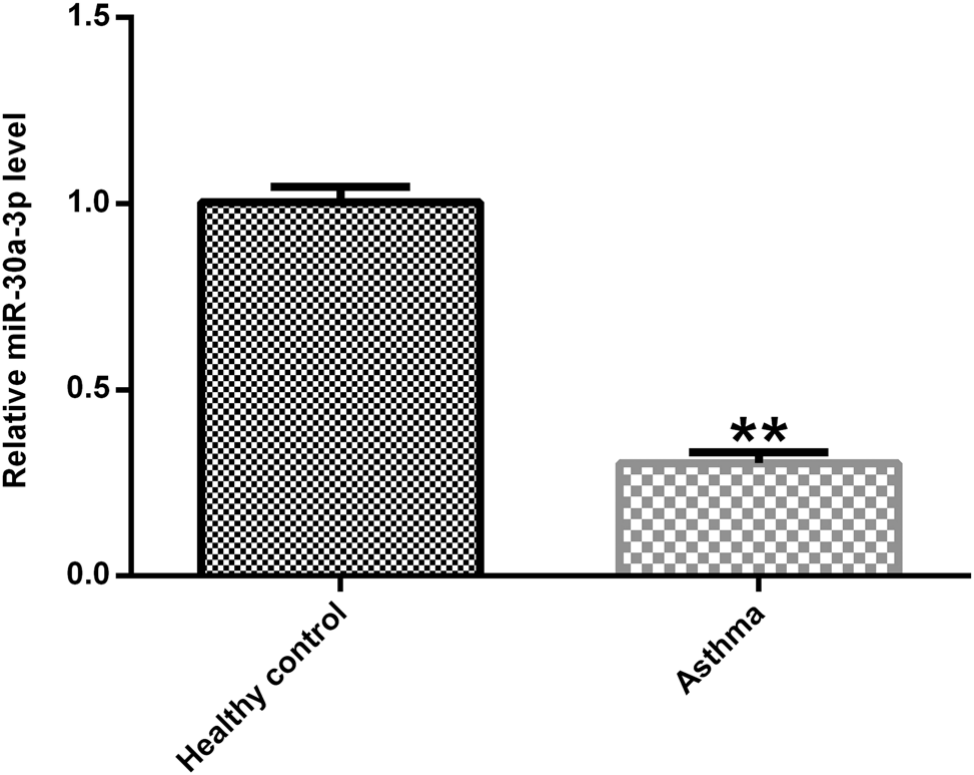
Expression of miR-30a-3p in asthmatic patients. The miR-30a-3p expression in asthmatic patients and healthy controls were determined using qRT-PCR.

Peripheral blood samples were collected from 30 asthmatic patients and 30 healthy volunteers. The miR-30a-3p expression was measured using qRT-PCR. The results revealed that miR-30a-3p expression was significantly downregulated in asthmatic patients compared with that noted to healthy volunteers (Figure 1).

### 3.2 CCR3 is a direct target gene of miR-30a-3p

**Figure 2:**
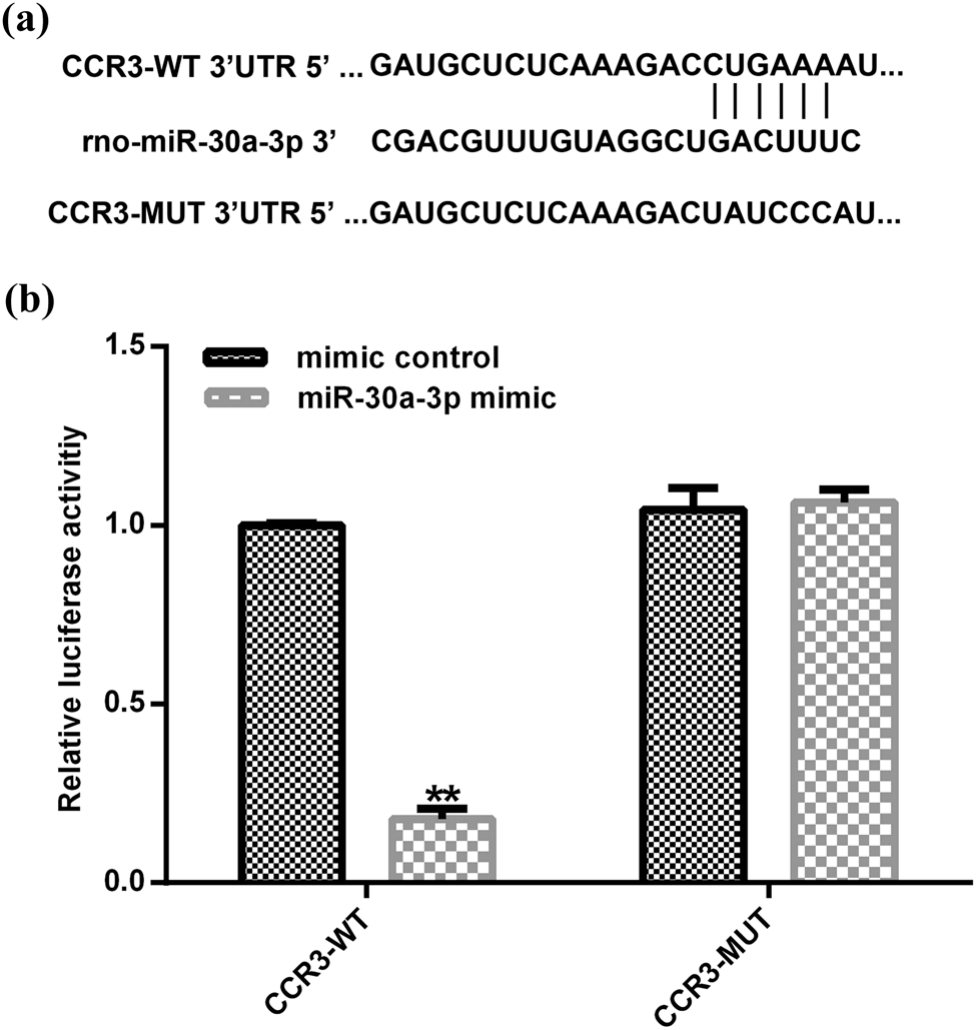
CCR3 is a target gene of miR-30a-3p. (a) The interaction between miR-30a-3p and 3′-UTR of CCR3 was predicted using a microRNA target site prediction software. (b) The direct targeting of miR-30a-3p to the 3′-UTR of CCR3 was verified using a dual-luciferase reporter assay. CCR3, CC chemokine receptor 3; 3′-UTR, 3′-untranslated region.

A target gene downstream of miR-30a-3p was identified using the TargetScan bioinformatics tool in order to determine the molecular mechanism of miR-30a-3p in asthmatic patients. Bioinformatic analysis showed that CCR3 was a direct target gene of miR-30a-3p (Figure 2a). In addition, bioinformatic predictions indicated that miR-30a-3p was partially complementary to the 3′-UTR of CCR3. Subsequently, eosinophils were co-transfected for 48 h with CCR3-WT, CCR3-MUT, miR-30a-3p mimic, or negative control. Dual-luciferase reporter assay was performed in order to detect the activity of luciferase and the results indicated that miR-30a-3p mimic could inhibit the activity of CCR3-WT but not that of CCR3-MUT (Figure 2b).

### 3.3 Expression of chemokines and Th2 cytokines in a mouse model of asthma

**Figure 3:**
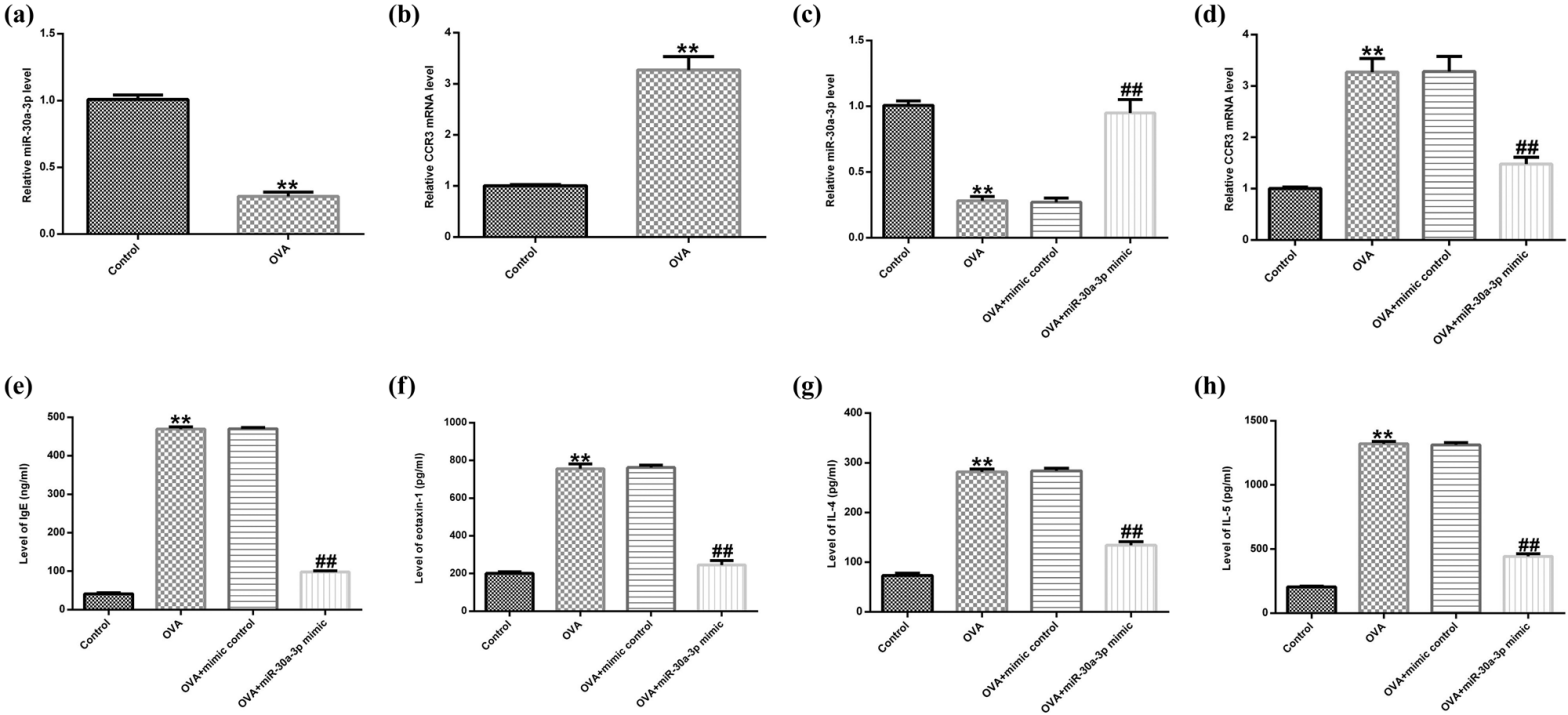
MiR-30a-3p decreases the expression of chemokines and Th2 cytokines. (a and c) The expression of miR-30a-3p was determined using qRT-PCR. (b and d) The CCR3 gene expression in different groups was determined using qRT-PCR. The secretion levels of (e) OVA-specific IgE, (f) IL-4, (g) IL-5 and (h) eotaxin-1 were determined using ELISA assay. Th2, type 2; CCR3, CC chemokine receptor 3; OVA, ovalbumin.

An asthmatic mouse model was established and the expression levels of miR-30a-3p were detected in blood using qRT-PCR. The results showed that the expression levels of miR-30a-3p (Figure 3a) and CCR3 (Figure 3b) were significantly downregulated and upregulated, respectively, in the peripheral blood of asthmatic mice compared with those noted in the control group. Subsequently, asthmatic mice were treated with intraperitoneal injection of miR-30a-3p mimic or mimic control. MiR-30a-3p and CCR3 expression levels were upregulated and downregulated, respectively, in the OVA + miR-30a-3p mimic group compared with the corresponding levels noted in the control group (Figure 3c and d). In addition, the serum levels of OVA-specific IgE, IL-4, IL-5 and eotaxin-1 in the OVA-induced mouse model were significantly increased, whereas treatment with miR-30a-3p significantly reduced their serum levels (Figure 3e–h). The aforementioned results suggested that miR-30a-3p was downregulated in asthma and CCR3 was a direct target gene of miR-30a-3p. Therefore, miR-30a-3p could inhibit CCR3 signaling pathway and alleviate Th2 cytokine production in asthmatic mice.

### 3.4 Effects of miR-30a-3p mimic on eosinophil biological behavior

**Figure 4:**
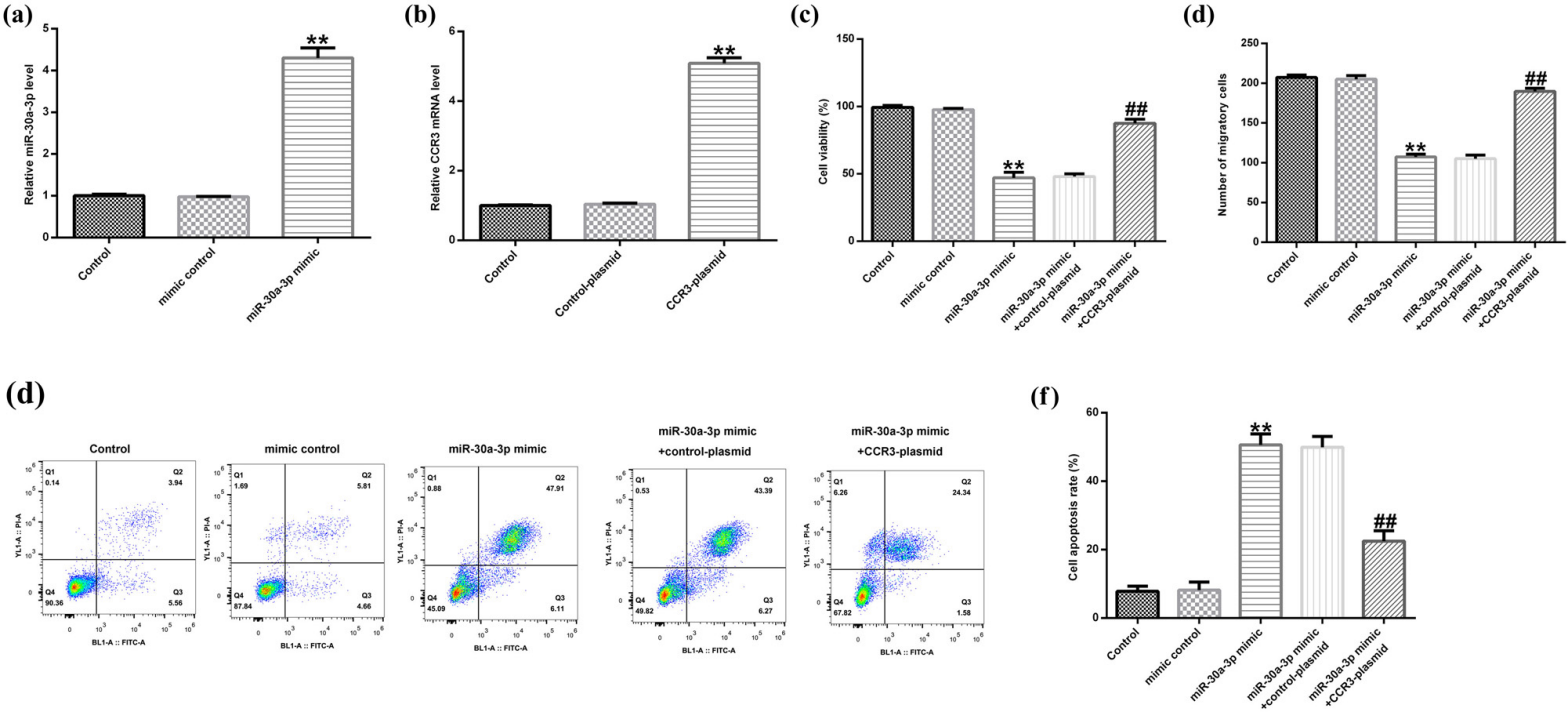
The effect of miR-30a-3p mimic on the biological behavior of eosinophils. The gene expression levels of (a) miR-30a-3p and (b) CCR3 were determined using qRT-PCR. (c) Cell viability was determined using CCK-8 assay. Eosinophils were transfected with miR-30a-3p mimic, mimic control, miR-30a-3p mimic + control-plasmid, or miR-30a-3p mimic + CCR3-plasmid. (d) Cell migration was determined using transwell assay. (e and f) Cell apoptosis was determined using flow cytometry assay. CCR3, CC chemokine receptor 3.

Eosinophils were transfected with miR-30a-3p mimic or mimic control for 48 h and qRT-PCR was performed. The results indicated that miR-30a-3p mimic significantly increased the expression levels of miR-30a-3p in mouse eosinophils (Figure 4a). Subsequently, the cells were transfected with CCR3-plasmid or control-plasmid. CCR3 expression was upregulated in the CCR3-plasmid group compared with that noted in the control-plasmid group (Figure 4b). In addition, CCK-8, transwell, and flow cytometry assays were performed in order cell viability, migration, and apoptosis, respectively, to be assessed. MiR-30a-3p mimic significantly reduced eosinophil cell viability compared with that of the control group (Figure 4c). Similar results were noted for migratory ability (Figure 4d), while induction of cell apoptosis was also evident (Figure 4e and f). The changes in the viability, migratory ability and apoptosis rate of eosinophils were significantly reversed by transfection of the cells with CCR3-plasmid.

### 3.5 Effect of miR-30a-3p mimic on CCR3 and eotaxin-1 expression in eosinophils

**Figure 5:**
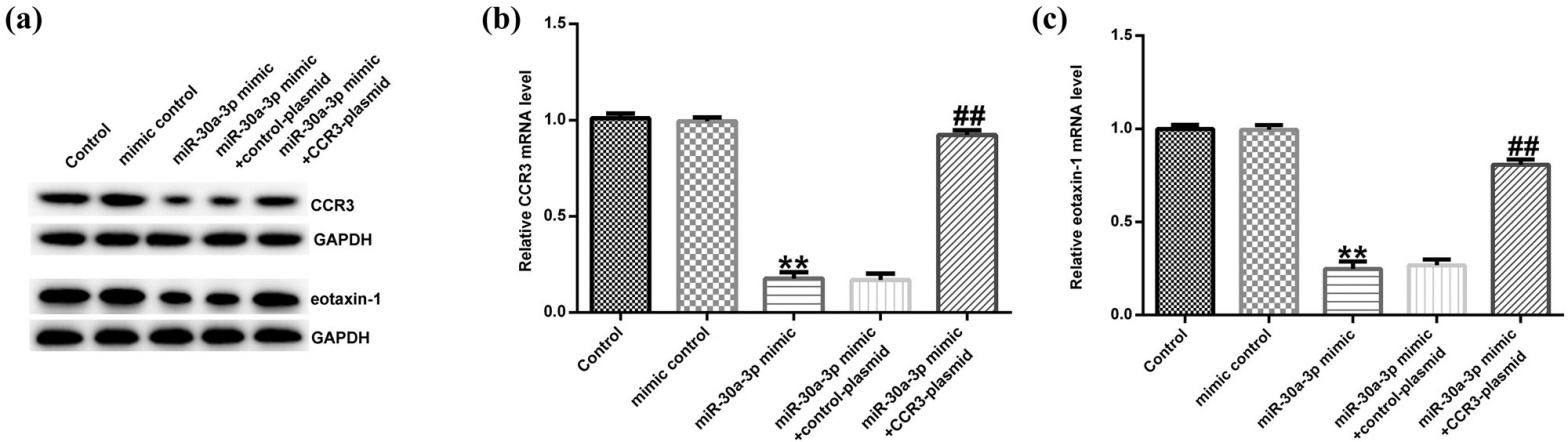
The effect of miR-30a-3p mimic on the CCR3 signaling pathway in eosinophils. Eosinophils were transfected with miR-30a-3p mimic, mimic control, miR-30a-3p mimic + control-plasmid, or miR-30a-3p mimic + CCR3-plasmid for 48 h. (a) The protein expression levels of CCR3 and eotaxin-1 were determined using western blot assay. (b and c) CCR3 and eotaxin-1 gene expression levels were determined using qRT-PCR. CCR3, CC chemokine receptor 3.

The effect of miR-30a-3p on CCR3 and eotaxin-1 expression was subsequently investigated. Mouse eosinophils were transfected with miR-30a-3p mimic + control-plasmid or miR-30a-3p mimic + CCR3-plasmid, and western blot and qRT-PCR assays were performed. CCR3 and eotaxin-1 protein (Figure 5a) and mRNA (Figure 5b) levels were significantly reduced in eosinophils transfected with miR-30a-3p mimic. In contrast to these findings, CCR3 and eotaxin-1 downregulation was reversed following CCR3-plasmid transfection of the cells (Figure 5c).

## 4 Discussion

Increasing evidence has indicated that the incidence and mortality rates of asthma have markedly increased worldwide. It has been reported that glucocorticoid drugs exhibit protective effects on airway remodeling and inflammation in a mouse asthma model [32,33,34]. However, prolonged use of drugs can produce potentially severe adverse effects. Therefore, the development of novel drugs to treat asthma is urgently required. Accumulating evidence supports the notion that eosinophils act as effector cells that serve a crucial role in the development of asthma and allergic diseases [35]. In addition, the degree of eosinophilia in the inflammation site determines the severity of asthma and is associated with airflow limitations.

Previous studies have shown that miRNAs are involved in multiple key developmental pathways, whereas different miRNAs were associated with different diseases, such as inflammatory diseases, infections, developmental disorders, and cancer [36]. Yang et al. demonstrated that miR-30a-3p upregulation inhibited sepsis-induced cell proliferation [37]. In addition, miR-30a-3p/5p downregulation increased cell proliferation in esophageal squamous cell carcinoma [38]. Wang et al. indicated that miR-30a-3p suppressed cell proliferation and invasion in hepatocellular carcinoma (HCC) [19]. However, the role of miR-30a-3p expression in asthma development has not been previously reported. The results of this study suggested that miR-30a-3p was downregulated in asthmatic patients and miR-30a-3p could alleviate Th2 cytokine production in asthmatic mice. We also found that CCR3 was a direct target of miR-30a-3p.

The CC chemokine receptor 3 (CCR3), which is a cell surface guanosine-binding protein-coupled receptor encompassing a typical motif of seven hydrophobic regions, is primarily expressed on the cell surface of eosinophils [39]. CCR3 is activated in response to eosinophil chemokines leading to a G protein-dependent intracellular signaling cascade that ultimately results to the migration of eosinophils [40,41]. In patients with allergic diseases such as asthma, eosinophils are recruited into the lungs and hyperactivated at the site of inflammation. Therefore, eosinophils in allergic diseases serve as a histological marker and one of the major effector cell types leading to their pathology. In addition, the CCR3 signaling pathway is one of the key adjustment pathways that is involved in the recruitment and migration of eosinophils into the affected tissues. Therefore, it was hypothesized that miR-30a-3p may be involved in the development and progression of asthma via targeting the CCR3 signaling pathway and regulating eosinophil activity. In this study, miR-30a-3p mimic significantly decreased eosinophil viability and migration, and induced apoptosis. However, these biological functions were reversed by transfection of the cells with CCR3-plasmid. Wardlaw et al. demonstrated that antagonists of IL-5 and CCR3 suppressed eosinophil recruitment in allergic diseases [42]. Therefore, this study suggests that CCR3 is an important target in chronic allergic airway diseases. However, miR-30a-3p has many other targets, such as DNMT3a and MAD2L1, which were not investigated in this study. This was a limitation of the current study and further research is needed in future research.

In conclusion, the findings suggested that miR-30a-3p mimic significantly reduced eosinophil viability and migration and induced apoptosis via targeting CCR3, indicating that miR-30a-3p might participate in the development and progression of asthma through regulating eosinophil activity. The specific mechanism of miR-30a-3p involved in the occurrence and development of asthma needs further exploration.
